# A Knock-Out Blow

**Published:** 1897-12-18

**Authors:** 


### A KNOCK-OUT BLOW.

The recent death of a young man as the result of injuries received in a boxing match at the National Sporting Club raises questions of considerable interest both from a sporting and a medical legal point of view.

In this case all the evidence went to show that the deceased was in good condition up to the last round. In this round, however, he received two blows closely following one another, by which he was knocked down not to rise again. The first of these, according to the evidence of Sir George Chetwynd, was "in the pit of the stomach and above the belt," and this was followed up at once by "a blow with the right on the point of the chin, which caused him to fall backwards on his head." As soon as he fell "he lay there and did not move." He was carried upstairs and put to bed, and was attended by a doctor and a nurse, but next morning he died, never having recovered consciousness. At the post-mortem examination the internal organs were found to be perfectly healthy, except for signs of extensive old pleurisy on the left side. The brain itself was healthy, and there was no laceration of the brain substance, but "on the right side at the base of the skull there was a fracture about 1¼ inch long. There was an effusion of blood behind the surface of the brain corresponding with this fracture." Death was attributed to the fracture of the skull, and it was also stated that "the fracture might have been due to the blow on the chin or the fall on the back of the head." This, of course, leaves open the very point of interest, and it will not do to be too ready to charge the injury to the fall, for what we believe is technically spoken of as the "knock-out" is itself a very serious matter, often as it is recovered from.

In discussing the actual cause of death in those cases in which men are "knocked out" in the course of a fight, many points have to be borne in mind. A direct blow on the pit of the stomach, even above the belt, may cause a complete cessation of the action of the heart, which may or may not be recovered from, or, on the other hand, it might cause actual rupture of the heart. In this particular case these causes were put out of court by the post-mortem examination, although, in view of the skill shown up to that round in warding off the attacks of his antagonist, it is more than probable that the blow on the "pit of the stomach" so staggered the deceased as to prevent him guarding the "knock-out" which he received on the point of the chin.

But was the fracture and the effusion the result of this blow or of the injury received in falling? The evidence on this point was not conclusive; yet it is a matter of very considerable interest in regard to the disputed question as to the cause of the unconsciousness which follows on a well planted "knock out"—an unconsciousness which is not only absolute and complete but is sometimes associated with such an interference with the action of the heart as to make it plain that these cases, although they usually come round in a little time, have been within an ace of going out altogether. It has been thought that this condition is due to inhibition of the heart arising from disturbance of the pneumogastic nerve; but the fact that where death has ensued post mortem examination has sometimes shown physical changes at the base of the skull, makes it probable that in many cases the cause should be sought within the cranium, the force of the blow being transmitted through the jaw to the cranial bones. Two cases have occurred within the last few years in which hæmorrhage has been found within the skull after a "knock out" blow on the jaw, in one case being due to rupture of the lateral sinus, and in another being merely described as "cerebral hæmorrhage," In one of these instances the accident happened in the course of what is described as a "friendly sparring bout" between university students, so that with the evidence before us, we must not be too sure that even in this case the death, although quite properly returned as "accidental," was not the result of the blow rather than the fall.

We will not enter upon the sporting question as to the permissibility of this character of blow. The suggestion that when a man is sure of his "points" he would lie snug, to avoid further punishment, did not failure to come up to time involve loss of the match, seems hardly a worthy one; still, it may be true. In any case, it can hardly be denied that the possibility of at any moment winning a losing game by getting in the "knock-out" must tend to encourage undue violence as a match draws to a close.

